# Integrative measurement analysis via machine learning descriptor selection for investigating physical properties of biopolymers in hairs

**DOI:** 10.1038/s41598-021-03793-9

**Published:** 2021-12-21

**Authors:** Ayari Takamura, Kaede Tsukamoto, Kenji Sakata, Jun Kikuchi

**Affiliations:** 1 RIKEN Center for Sustainable Resource Science, 1-7-22 Suehiro-cho, Tsurumi-ku, Yokohama, Kanagawa 230-0045 Japan; 2 Graduate School of Medical Life Science, Yokohama City University, 1-7-29 Suehiro-cho, Tsurumi-ku, Yokohama, Kanagawa 230-0045 Japan; 3 Graduate School of Bioagricultural Sciences, Nagoya University, 1 Furo-cho, Chikusa-ku, Nagoya, Aichi 464-0810 Japan

**Keywords:** Solid-state NMR, Cheminformatics

## Abstract

Integrative measurement analysis of complex subjects, such as polymers is a major challenge to obtain comprehensive understanding of the properties. In this study, we describe analytical strategies to extract and selectively associate compositional information measured by multiple analytical techniques, aiming to reveal their relationships with physical properties of biopolymers derived from hair. Hair samples were analyzed by multiple techniques, including solid-state nuclear magnetic resonance (NMR), time-domain NMR, Fourier transform infrared spectroscopy, and thermogravimetric and differential thermal analysis. The measured data were processed by different processing techniques, such as spectral differentiation and deconvolution, and then converted into a variety of “measurement descriptors” with different compositional information. The descriptors were associated with the mechanical properties of hair by constructing prediction models using machine learning algorithms. Herein, the stepwise model refinement via selection of adopted descriptors based on importance evaluation identified the most contributive descriptors, which provided an integrative interpretation about the compositional factors, such as α-helix keratins in cortex; and bounded water and thermal resistant components in cuticle. These results demonstrated the efficacy of the present strategy to generate and select descriptors from manifold measured data for investigating the nature of sophisticated subjects, such as hair.

## Introduction

A scientific measurement provides information regarding a subject based on analytical principles. Efficient extraction and integration of various types of measured information is an ultimate interest in scientific analysis to comprehensively understand the nature of a subject. However, such data integration could be difficult if a subject is complicated; if multiple measurements are conducted complementarily; and if various complicated information are involved in the measured data.

A polymer is one of the most challenging analytical subjects since it is composed of a huge number of atoms and the assembled or higher-order structure is also crucial to determine the net properties. Such complicated polymer structures could not be assessed by previous computational descriptors which only represent “monomer” or “oligomer” structures based on atomic composition, molecular dynamic simulation^1^, quantum chemical calculation^2^, string notation (e.g., SMILES), and graph representation^3–6^. In contrast, experimental investigations of polymers can be conducted by various analytical techniques that target primary, secondary, higher-order, and large-scale structures, respectively. Therein, the association techniques of such manifold data are worth exploring in order to reveal the origins or compositional factors of polymers’ properties. In this study, we investigate the analytical strategies to extract and integrate the information measured by various analytical techniques, applying to sophisticated biological polymers derived from hair.

Hair is mainly composed of keratin fibers^7^. A small amount of lipids, water, and pigments are also present in hair^8^. Hair exhibits distinctive properties of high flexibility and high mechanical- and thermal- resistance. Histologically, hair consists of two structures: cortex (85–90%)^9,10^ and cuticle (around 10%)^11,12^. Cortex is the dominant inner material of hair, wrapped by cuticle and composed of an assembly of spindle-shaped macrofibrils. The macrofibrils have two main compositions: intermediate filaments (IF) and surrounding matrix, called intermediate filament associated proteins (IFAP)^7,13,14^. IF is composed of bundles of keratin fibers that form crystalline α-helical coiled-coil structures^15,16^. IFAP has amorphous matrix components with a relatively high amount of cysteine and, thus, contributes to the stabilization of the IF structure via disulfide linkage^7,17,18^. On the other hand, cuticle is the outermost layer and is composed of overlapping flattened cells, like scales, with a total thickness of ~ 5 μm^14,19^. Keratin fibers in cuticle are also amorphous and are rich in disulfide linkage, which is responsible for the chemical and mechanical resistance of hair^7,11,18,20^.

Hair has been studied on different structure levels for many years. At the level of monomer molecules, the amino acid composition of each hair component has been studied using liquid chromatography and color tests^8,10,21,22^. The establishment of isolation techniques for cortex and cuticle has also assisted these studies^20^. Vibrational spectroscopy, such as Fourier transform infrared (FT-IR) spectroscopy^23,24^ and Raman scattering^25–28^, has been helpful to understand conformation, oxidation state, and interaction with keratin surroundings. Nuclear magnetic resonance (NMR) spectroscopy has been a powerful tool in revealing the higher-order structure and mobility of the keratin complex, as well as molecular-level compositions^9,11,15,28,29^. X-ray diffraction has demonstrated, from molecular conformation to organization of the secondary structure of hair components^9,13,30^. Thermal analysis, such as differential scanning calorimetry and thermogravimetric analysis, has shown the thermal behaviors and resistance of different hair components according to their molecular compositions and higher-order structures^11,31^. As for the macrostructure, early histological observations have demonstrated fine layers in the cuticle^19^, hair diameter, and medullary index^32^ via transmission electron microscopy and scanning electron microscopy. These previous studies have provided multiphasic perspectives about hair structure and have indicated the sophisticated properties. Thus, integrative interpretation via different analytical techniques is required to deepen understanding of hair’s properties, but has not been achieved yet.

The main objective in this study is to demonstrate an analytical strategy to discover the most correlative information from various measured data to physical properties of biopolymers, which then enables integrative interpretations between them. Herein, we acquired the measured data of hairs by multiple analytical techniques, including solid-state NMR, time-domain (TD)-NMR, attenuated total reflection (ATR) FT-IR spectroscopy, and thermogravimetric and differential thermal analysis (TG–DTA). Different data-processing techniques, such as binning, spectral differentiation, dimension reduction, and curve deconvolution, were applied to the measured data. Then, a variety of “measured descriptors” were generated to involve different measured information, such as molecular compositions, morphologies and mobilities^33–38^. At the same time, a tensile tester was used to evaluate physical properties of hair, such as breaking force, elastic modulus, extension, and yield strength. These physical properties were expected to depend on molecular composition and morphology, as well as the histological architecture, of hair. Subsequently, the relationship between the generated measured descriptors and the physical properties were investigated by constructing predictive models based on machine-learning algorithms. Herein, evaluation of the “importance” of each descriptor were utilized to select more contributive ones for predicting respective physical properties. Rigorous stepwise selection of the descriptors adopted achieved to determine the most contributive descriptor sets for respective physical properties via two different machine-learning algorithms. Consequently, the selected measured descriptors enabled integrative interpretation about the compositional origins of the physical properties. In addition, this study demonstrated the efficacy of the present descriptor selection strategy as well as the data-processing to generate the measurement descriptors for integrative analysis.

## Experimental

### Hair samples

Hair samples in the present study were collected from cats, cows, humans, and pigs. The species were chosen to cover diverse ranges of physical properties. Nine cat hair pools were acquired from two domestic cats brushed on different days. Twelve cow hair pools were provided from Sermas Co. Ltd. (Chiba, Japan). Twenty-one human hair pools were collected from 10 volunteers, cut on different days. Twenty-one pig hair pools were provided by Sermas Co. Ltd. (Chiba, Japan). Each sample pool was composed of hair collected from individual donors. The hairs were stored in a desiccator at room temperature of 22 °C, in specified rooms, until used. All experimental procedures involving animals and human subjects were approved by the Institutional Ethics Committee of RIKEN Yokohama Branch and carried out in accordance with relevant guidelines and regulations^39^. Informed consent was obtained from all human donors and a legal guardian, as well as owner and volunteer for the animal studies.

### Evaluation of physical properties

Cross-section areas of hairs from the individual hair pools were evaluated based on the diameter gauged by a micrometer. Mechanical physical properties (i.e., breaking force, elastic modulus, extension, and yield strength) of the hairs were evaluated using a tensile tester (EZ-L-5 kN; Shimadzu Co. Ltd., Japan). Single hair fibers with lengths of 10 mm were tested at a strain rate of 30 mm/min. The software included with the tester (TRAPEZIUM2 ver. 2.36; Shimadzu Co. Ltd.) was used to operate the instrument and to calculate each physical property based on the measured stress–strain curves and hair cross-section areas. Ten hair fibers from each sample pool were subjected to the measurements, and averaged values and the standard deviations of the respective physical properties were obtained.

### Measurements

Solid-state NMR spectra were recorded on a DRX-500 spectrometer (Bruker-BioSpin, MA, USA), equipped with a Bruker MAS VTN 500SB BL4 probe. The spectrometer frequency was 500.132 MHz for ^1^H NMR spectra and 125.758 MHz for ^13^C NMR spectra. The instruments were operated by TopSpin 3.2 software (Bruker, MA, USA). Hair samples were measured at room temperature. ^1^H NMR spectra were recorded without and with magic angle spinning (MAS) at a rate of 12 kHz and designated as ^1^H wide-line (anisotropic) spectra and ^1^H MAS (isotropic) spectra, respectively. The dwell time was 2.5 μs for ^1^H wide-line spectra and 20 μs for ^1^H MAS spectra. The dead time was 6.5 μs, and the recycle delay was 5 s for all ^1^H NMR spectral measurements. ^13^C NMR spectra were measured using cross polarization (CP)-MAS with a contact time of 1.2 ms and MAS rate set to 12 kHz. ^13^C chemical shifts were externally referenced to the glycine carbonyl signal at 176.03 ppm.

TD-NMR spectra of hairs were measured using a Minispec mq20 NMR spectrometer (Bruker, MA, USA) at 298 K. The instrument was operated at a ^1^H frequency of 19.9 MHz (0.5 T) and equipped with a VT temperature control system operating with nitrogen gas. A standard solid echo pulse sequence was used with a dead time of 9.3 μs and π/2 pulse of 2.88 μs. The signal decays were recorded up to 1.0 ms. TD-NMR measurements were conducted five times for each hair pool.

FT-IR spectra were measured by an FT-IR spectrometer (Nicolet 6700 spectrometer, Thermo Fisher Scientific Inc., MA, USA) using an ATR accessory with a diamond crystal. The spectral range was 650–4000 cm^−1^ with a resolution of 4 cm^−1^. Each spectrum was acquired via scans run 16 times. At least three spectra were collected from each sample pool. The FT-IR instruments were operated using the included software (OMNIC; Thermo Fisher Scientific Inc.).

Prior to TG–DTA, hairs from individual sample pools were crushed by a freezing-crushing device for 5 min. A 10–25 mg mass of the hair fragments was stuffed into an aluminum pan and then inserted into the thermogravimetric and differential thermal analyzer (EXSTAR TG/DTA 6300; SII Nanotechnology Inc., Japan). The thermogravimetry values were recorded from 40 to 500 °C, at a rate of 5 °C/min, under nitrogen flowed at 200 mL/min. Derivative thermogravimetry (DTG) curves (g/min) were provided by TA/7000 software (Hitachi Co., Japan).

### Pre-treatment of measured data

Prior to being processed into descriptors, all the raw measured data were subjected to consistent pre-treatment including profile-differentiation and normalization. The measured data by different analytical technique also needed specific pre-treatment depending on the data-property and data-ranges as follows.

For the solid-state NMR spectra, baseline and phase corrections were performed by MNova software (Mestrelab Research, Spain). The NMR spectra were subsequently processed using IGOR Pro software (WaveMetrics Inc., OR, USA). NMR spectra were aligned with consistent axes of chemical shift by cubic spine interpolation. Furthermore, second-order derivative NMR spectra were acquired using the third polynomial Savitzky–Golay method. The nonderivative and second-derivative NMR spectra were truncated so that they maintained the following informative spectral regions: − 102 to 102 ppm for the ^1^H wide-line spectra, − 8.0 to 14.2 ppm for the ^1^H MAS spectra, and 2.8–185.2 ppm for the ^13^C CP-MAS spectra. The NMR spectra were finally normalized by the total area.

The decay curves of TD-NMR were fitted as a combination of three components using the Abragamian function with TD-NMR Analyzer software ver. 7.0 (Bruker), calculating intensity proportion and the relaxation time of each component. The measured curves were then normalized so that the sum of the intensities of the fitted curves was one at time equal to zero seconds.

The FT-IR spectra were second-order differentiated by the third polynomial Savitzky–Golay method. The spectral regions of 1711–2669 and 3400–4000 cm^−1^ were excluded due to crystal interference^40–42^. Derivative FT-IR spectra were normalized by the total area. Finally, the averaged derivative FT-IR spectra were obtained based on the spectra collected from the respective hair pools.

The DTG curves acquired from 44 to 497 °C were first binned into the unit size of 1 °C. Then, the curve intensities were normalized by the sample weight, resulting in the unit of %/min. The DTG curves were further second-order differentiated by the third polynomial Savitzky–Golay method to enhance features.

### Generation of measurement descriptors

The pre-treated data were subsequently converted into “measurement descriptors” via two common processing techniques of binning and dimension reduction by principal component analysis (PCA). The ^1^H wide-line NMR spectra and TD-NMR decay curves were also applied to curve deconvolution analysis. All generated measurement descriptors are listed in Table S1 with detailed data information.

Binning of the nonderivative and second-order derivative profiles of the solid-state NMR spectra, the FT-IR spectra, and the DTG curves was performed for the data-regions involving significant signals as detailed in Table S1. The data-regions were split into series of small regions (i.e., bins) with even steps so as to keep the characteristic peaks resolved. The number of split bins for respective data were listed in Table S1. Meanwhile, the decay curves of TD-NMR were split into 40 bins with logarithmic steps up to 1.0 ms. Then, average value within each bin was calculated as a descriptor.

PCA was implemented for the respective datasets (i.e., the nonderivative and second-order derivative profiles of the solid-state NMR spectra, the FT-IR spectra, and the DTG curves; and pre-fitted and fitted decay curves of TD-NMR) after mean-centering. Proportions of variance of the principal components were calculated for each dataset. Corresponding scores of the principal components with a proportion of variance greater than 1% were adopted as descriptors.

Curve deconvolution was conducted for the ^1^H wide-line NMR spectra and TD-NMR decay curves. The ^1^H wide-line NMR spectra were decomposed into three peaks of a Voigt function using the multipeak fitting package in IGOR Pro software. Then, the area proportion and the full width at half maximum (FWHM) of each peak were obtained. Deconvolution of the TD-NMR decay curves was performed as described above, depending on the intensity proportion and the relaxation time of each component. The obtained area proportion and FWHM of the ^1^H wide-line NMR spectra and the intensity proportion and relaxation time of the TD-NMR decay curves were further processed into inverses, exponentials, logarithms, and mutual ratios. The values of the respective TD-NMR descriptors were obtained via averaging the results of five measurements for each sample pool.

### Selection of measurement descriptors for physical property prediction

Data analysis to associate measurement descriptors with physical properties was conducted using R software with the Rstudio environment. The relationship between generated descriptors and physical properties was first overviewed by canonical correlation analysis (CCorA) using the CCorA function in the R package “vegan.” The examined sets of measurement descriptors were determined such that the Pearson correlation coefficient of each set was less than 0.4 and 0.3 for individual measurement methods and the all-method, respectively. The datasets of physical properties and measurement descriptors were standardized before CCorA.

Prediction models for the physical properties were trained based on random forest (RF) or partial least squares regression (PLSR) algorithms. The RF models were built using the randomForest function in the R package, “randomForest,” and the number of trees to grow was set to 1000. The PLSR models were built using the plsr function in the R package: “pls.” The explanatory variables of the measurement descriptors (input) were standardized. The response variables of the physical properties (output) were mean-centered. The number of latent variables adopted in the PLSR models was determined so as to provide the minimum predicted residual error sum of squares evaluated by a ten-fold cross-validation (CV). The technique of ten-fold CV prepares ten randomly split sub-datasets. A model was trained using the nine sub-datasets, then validated by the one sub-datasets left. The procedures repeated time times, changing the sub-dataset used for validation. The regression errors of each model were evaluated based on the root mean squared error (RMSE) and coefficient of determination (*R*^2^) obtained through 100 repeats of the ten-fold CV. Adjusted *R*^2^ was not applied because neither RF nor PLSR algorithm relies on direct multiple regression with explanatory variables. In addition, CV avoids overestimation and variations of model performance evaluation. To find more contributive descriptors for the physical properties, the importance of each descriptor was evaluated using the randomForest function with an augment of “importance” for the RF models and the varImp function in the R package, “caret,” for the PLSR models and. The importance was averaged for 100 repeats of the ten-fold CV. The descriptors were sorted in decreasing order of averaged importance, and 90% of the higher-rank descriptors were then successively used in the next model training. Consequently, the descriptor set that provided the best prediction accuracy (i.e., the highest *R*^2^) was determined for each series of the prediction models.

## Results and discussion

### Physical properties and measurements of hair

**Figure 1:**
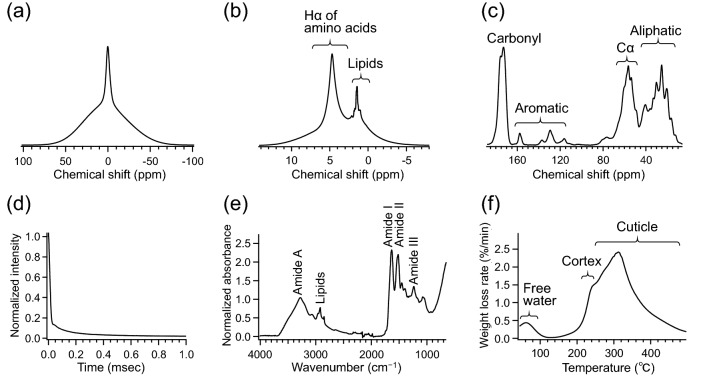
Measurements of hairs. Hair samples were subjected to (**a**) solid-sate NMR experiments to provide ^1^H wide-line spectra, (**b**) ^1^H MAS spectra, and (**c**) ^13^C CP-MAS spectra; (**d**) TD-NMR for magnetization decays, (**e**) FT-IR spectroscopy, and (**f**) TG–DTA to yield DTG curves. The averaged data after normalization are shown.

The hair samples collected from different species were analyzed by several measurement techniques: solid-state NMR, TD-NMR, FT-IR, and TG–DTA. For solid-state NMR, the ^1^H wide-line (anisotropic) spectra, ^1^H MAS (isotropic) spectra, and ^13^C CP-MAS spectra were recorded. Figure 1 shows the averaged data of each measurement. The ^1^H wide-line spectra exhibited the typical line shape of solid samples, which broadens over a range of 100 ppm due to the various orientations of dipolar interactions (Fig. 1a and Fig. S1a). At the same time, a relatively narrow peak was observed around 0 ppm. These line shapes in the ^1^H wide-line spectra indicated that hair samples contained compositions with different molecular mobilities, or anisotropic interaction^15^. Meanwhile, the ^1^H MAS spectra showed characteristic peaks within a narrower spectral region owing to averaged isotropic interactions by MAS (Fig. 1b). Some sharp peaks, from 0.8 to 2.3 ppm, were ascribed to lipid compositions^43,44^. The lipid peaks were the most distinct in cat hairs and hardly observed in pig hairs (Fig. S2a). The relatively broad peak around 2.8–7.0 ppm encompasses Hα of amino acids, which is mainly keratins^43,45^. In addition, the line shape widely expanding from − 5 to 14 ppm may represent highly anisotropic and rigid components, such as structured keratins. The ^13^C CP-MAS spectra exhibited distinctive peaks of side-chain aliphatic carbons, Cα methine carbons of amino acids, aromatic carbons, and carbonyls carbons around 10–40 ppm, 45–60 ppm, 115–158 ppm, and 165–178 ppm, respectively (Fig. 1c)^9,28,43,46–48^. The pig hairs showed a relatively higher intensity of carbonyl carbons, assignable to the α-helix form around 176 ppm, among the hair types (Fig. S3a)^9,28,46,47^. The signals observed by TD-NMR rapidly decayed at an earlier time, then gradually decreased to zero (Fig. 1d). The decay curves demonstrated the presence of compositions with different relaxation rates, or mobilities, in hairs^49^. This was consistent with the line shapes of the ^1^H wide-line NMR spectra. The TD-NMR curves of hairs showed a similar tendency for each species, whereas substantial donor-dependent differences were simultaneously involved (Fig. S4a). The FT-IR spectra also showed characteristic absorption peaks of proteins and lipids (Fig. 1e). Peaks of Amide A, Amide I, Amide II, and Amide III, derived from proteins, were observed around 3277, 1634, 1516, and 1234 cm^−1^, respectively^50–52^. Methyl and methylene stretching at 2958 and 2850 cm^−1^ were representative of lipids^53^. The hairs of each species showed similar spectral patterns with intensity variations, particularly at lipid peaks (Fig. S5a). TG–DTA provided DTG curves of the hair samples (Fig. 1f). The mass loss under 100 °C represented the removal of free water^54–56^. Distinct mass loss up to around 240 °C was considered pyrolysis of cortex according to previous reports^11,31^. After the pyrolysis of cortex, the remaining cuticle forms “micro-tubes” emptied of cortical material. Mass loss that follows should correspond to decomposition of the micro-tubes, which could possibly be preceded by the elimination of bound water^54,57^. The carbonization of the remaining constituents proceeded further until reaching the end temperature of 500 °C. The human hairs showed slightly higher values in DTG curves at around 240–260 °C than those of other species (Fig. S6a).

The hair samples were also subjected to a tensile tester to evaluate the following physical properties: breaking force, elastic modulus, extension, and yield strength. The averaged physical property values of each hair sample are plotted with the standard deviations in Fig. S7. The breaking force was high for pig hairs (median of 6.02 N) and relatively low for cat hairs (median of 0.21 N), which were well correlated with the cross-section areas (Fig. S7a). Meanwhile, cow hairs demonstrated relatively high elastic modulus (median of 4.6 GPa) (Fig. S7b), and human hairs showed a bit higher extension (median of 65%) (Fig. S7c) among tested hair types. Yield strength among tested hair types was relatively low for cat (99 MPa) and human hairs (105 MPa), whereas it was high for cow hairs (177 MPa) (Fig. S7d). Owing to the characteristic properties depending on species, as well as individual donors, the collected hair samples provided a substantial variety of physical property values. Here, it should be noted that because of intrinsic biological variations within each hair sample pool, the observed values showed considerable variations, resulted in the substantial standard deviations (Fig. S7).

### Generation of measurement descriptors

**Figure 2:**
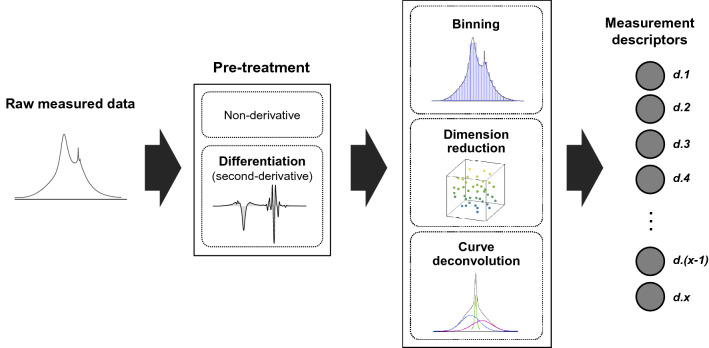
Schematic of development of measurement descriptors. The raw measured data of hairs were pre-treated with or without differentiation. The pre-treated data were subsequently subjected to the processing of binning, dimension reduction, or curve deconvolution. Then, a variety of “measurement descriptors” were generated from the data measured by different analytical techniques: a total of 902 descriptors.

The measurement data of hairs were converted into “measurement descriptors” by the data-processing, including spectral differentiation, binning, dimension reduction by PCA, or curve deconvolution (Fig. 2). Second-order differentiation was applied to the ^1^H wide-line, the ^1^H MAS, ^13^C CP-MAS NMR spectra, the FT-IR spectra and the DTG curves in order to enhance the profiles’ features. The differentiation is also effective to correct offset or linear drift of baseline. Binning was conducted to calculate the average values within certain regions in the profiles so as to represent the characteristic peaks as resolved. Dimension reduction aimed to extract correlating variable sets for representing the data’s features efficiently. Curve deconvolution for the ^1^H wide-line NMR spectra and the decay curves of TD-NMR was separation of the mixed signals into a small number of components via function fitting. Schematics of the generated bins and deconvoluted components are shown, along with their respective measurement results, in Figs. S1–S6. Consequently, a total of 902 descriptors were generated. All measurement descriptors are detailed in Table S1.

**Figure 3:**
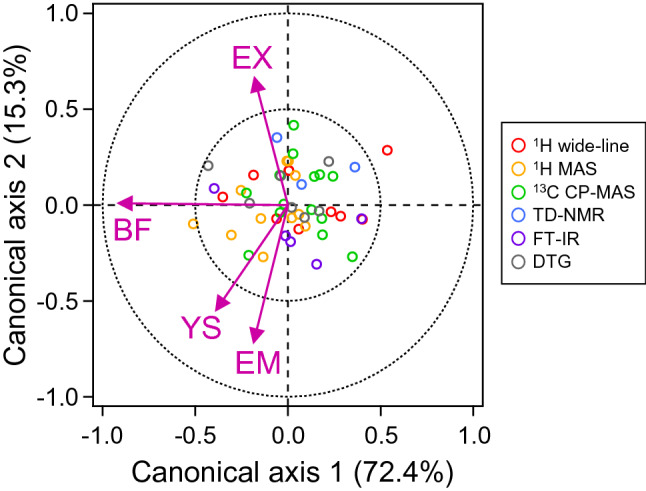
Canonical correlation analysis between physical properties and measurement descriptors of hairs. Datasets of the measurement descriptors were prepared from all experiments using a correlation less than 0.3. Datasets of the physical properties and measurement descriptors were standardized in advance. Calculated scores for the descriptors of the ^1^H wide-line, ^1^H MAS, and ^13^C CP-MAS NMR spectra; TD-NMR and FT-IR spectra, and DTG curves were plotted with open dots of red, orange, green, blue, purple, and black, respectively. Scores for the physical properties of breaking force (BF), elastic modulus (EM), extension (EX), and yield strength (YS) are represented with solid arrows.

To overview the relationship between the generated descriptors and physical properties, CCorA was conducted. CCorA determines a set of linear combinations of variables in two datasets (i.e., physical properties and measurement descriptors) so as to maximize the correlation between them^58^. CCorA results were obtained for descriptor sets of each measurement (Fig. S8) and the combined set (Fig. 3). Breaking force was plotted with a relatively large score (~ 1) on the first, or the most dominant, canonical axes in all plots. This tendency demonstrated that breaking force was well explained by the prepared descriptors. On the other hand, elastic modulus, extension, and yield strength were expressed mainly on the second canonical axis in most plots. In addition, elastic modulus and yield strength were plotted close to each other. This result shows that these two properties have similar correlation with measured data. Meanwhile, extension was plotted on the opposite side of the plot, indicating different and distinctive correlation with the measurements (Fig. 3). Relative contributions of the measured information to physical properties were difficult to compare based on these CCorA results. However, some of the less-promising descriptors indicated by small scores for physical properties were descriptors of ^1^H wide-line NMR spectra for extension (Fig. S8a) and descriptors of ^1^H-MAS NMR spectra for elastic modulus (Fig. S8b).

### Prediction of physical properties by measurement descriptors

The measurement descriptors were further associated with physical properties by building prediction models using RF and PLSR, which are nonlinear and linear algorithms, respectively. Herein, each of the physical properties (output) was predicted by the measurement descriptors (input) generated from their respective or all of the measurements. The constructed models were validated by ten-fold CV. The ten-fold CV was repeated 100 times, and the averages and standard deviations of RMSE and *R*^2^ were then calculated (Table S2). According to the results of CCorA, breaking force was predicted accurately with a high *R*^2^ of ~ 0.913; meanwhile, the predictions for elastic modulus, extension, and yield strength showed relatively poor accuracies or no significant correlations (*R*^2^ < 0.4). The descriptor set combined from all the measurements was expected to provide superior predictions using multiple types of measured information. However, the prediction accuracies obtained by the combined descriptor set were comparable or a bit poorer than those by the descriptors from each measurement. This result indicated that the presence of uncorrelated measurement descriptors in the explanatory variable set possibly hindered efficient prediction, making the integrative interpretation difficult. Therefore, selection of the descriptors adopted for the predictive modeling were requisite to improve the prediction accuracy, then determine the ones with significant contributions to the physical properties.

### Selection and interpretation of measurement descriptors

**Figure 4:**
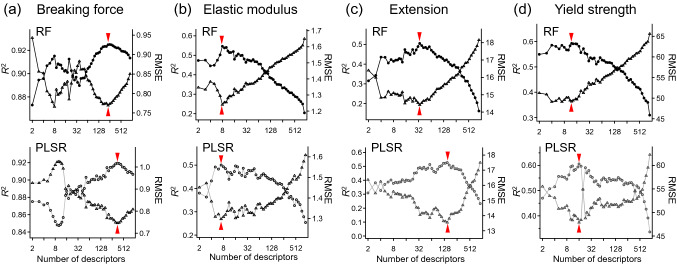
Selection of measurement descriptors for physical property prediction of hairs. Series of the prediction models were constructed for (**a**) breaking force, (**b**) elastic modulus, (**c**) extension, and (**d**) yield strength using random forest (RF) (top, black markers) and partial least squares regression (PLSR) (bottom, gray markers). The number of adopted descriptors was reduced stepwise from a total of 902. The prediction accuracies of *R*^2^ (circles) and RMSE (triangles) were evaluated at each step. The best results with the highest *R*^2^ are indicated with red arrow heads for each model series.

**Figure 5:**
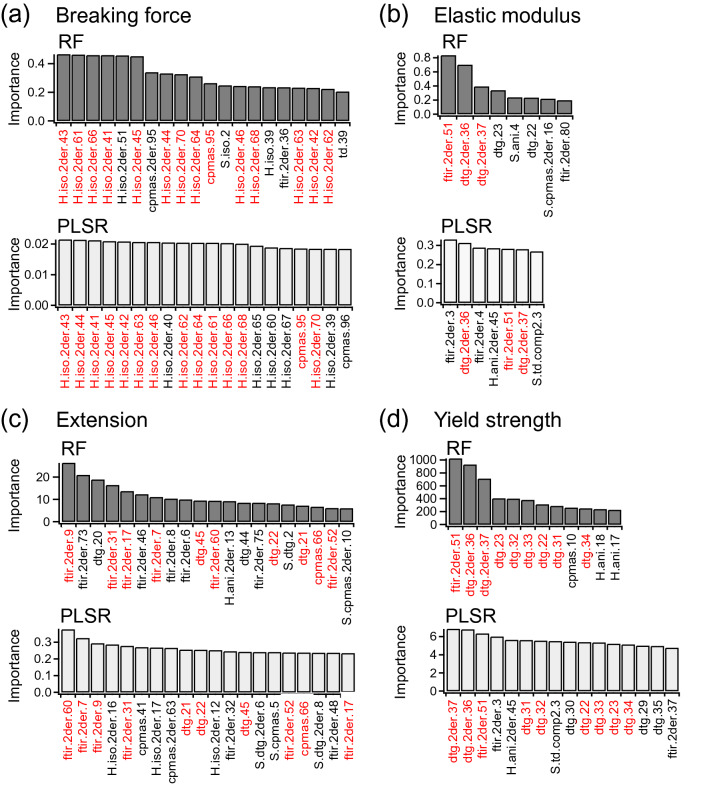
Measurement descriptors selected to predict physical properties of hairs. The 20 best descriptors selected using random forest (RF) (top, black bars) and partial least squares regression (PLSR) (bottom, gray bars) are listed with the evaluated importance for (**a**) breaking force, (**b**) elastic modulus, (**c**) extension, and (**d**) yield strength. The descriptors commonly selected by RF and PLSR are highlighted with red.

To realize reliable integrative interpretation from multiple measured information, sufficient correlations by predictive modeling are expected. Thus, selection of contributive measurement descriptors from all 902 generated was subsequently performed, aiming to improve model’s performance. When building a RF or PLSR model, the importance of each measurement descriptor was evaluated via 100 repeats of ten-fold CV. Followingly, 90% of measurement descriptors ranked with higher importance values were used for the next model building, then the number of the descriptors adopted was reduced stepwise. Prediction accuracies (i.e., RMSE and *R*^2^) of the RF and PLSR models built at each step are shown for their respective physical properties (output) in Fig. 4. As a general trend, starting from 902 descriptors, *R*^2^ values first increased (and RMSE decreased), then reached the maxima. This process should correspond to elimination of insignificant descriptors. Further reduction of the number of the descriptors resulted in the decrease of the *R*^2^ values, indicating that the contributive descriptors were excluded. Consequently, the refined descriptor sets that showed the highest *R*^2^ values were determined to be the best among each selection series. At the same time, the *R*^2^ values of more than 0.5 were assured for the significant correlations. Fig. S9 shows plots of predicted physical properties values with the best descriptor sets versus observed values. As a result, the RF and PLSR models for each physical property showed common descriptors of the 20th-best importance (Fig. 5). Such descriptors commonly selected by two different algorithms indicated the reliable descriptors selection based on the importance evaluation, and would be especially useful for interpretations of the association with the physical properties. As tendencies, breaking force greatly relied on the descriptors of ^1^H MAS and ^13^C CP-MAS NMR spectra; the predictions of elastic modulus, extension, and yield strength were mainly attributed to descriptors of the FT-IR spectra and DTG curves. Moreover, the descriptor selection process significantly contributed to decreases of the prediction errors (Fig. 4 and Table 1). Even though the observed physical properties themselves involved considerable variations within each hair sample (Fig. S7), the RMSE obtained in the best models were significantly lower than the experimental standard deviations and the *R*^2^ exceeded 0.5 (Table 1). This result represents that the developed predictive models substantially reflected the correlation between the observed physical properties. At the same time, not-excessive *R*^2^ indicates a result from avoiding over-fitting to the experimental errors in the observed physical properties as well as the respective measurements.

**Table 1:** Prediction of physical properties using sets of measurement descriptors selected as the best.

Physical property (output)	Experimental standard deviation^a^	RF			PLSR		
		No. of selected descriptors (input)	RMSE^b^	*R*^2 b^	No. of selected descriptors (input)	RMSE^b^	*R*^2 b^
Breaking force (N)	1.182	255	0.773 ± 0.022	0.925 ± 0.005	349	0.745 ± 0.025	0.919 ± 0.005
Elastic modulus (GPa)	1.41	8	1.24 ± 0.089	0.546 ± 0.073	7	1.30 ± 0.052	0.500 ± 0.038
Extension (%)	21.5	34	14.4 ± 0.3	0.506 ± 0.027	150	13.5 ± 0.3	0.527 ± 0.021
Yield strength (MPa)	59.4	12	49.1 ± 1.7	0.593 ± 0.030	16	47.8 ± 1.3	0.606 ± 0.022

^*a*^Mean experimental standard deviation of observed physical property was calculated as square root of average of experimental variances in ten times of testing each hair sample pool.

^*b*^Each value was evaluated by 100 repeats of ten-fold CV (average ± standard deviation).

**Figure 6:**
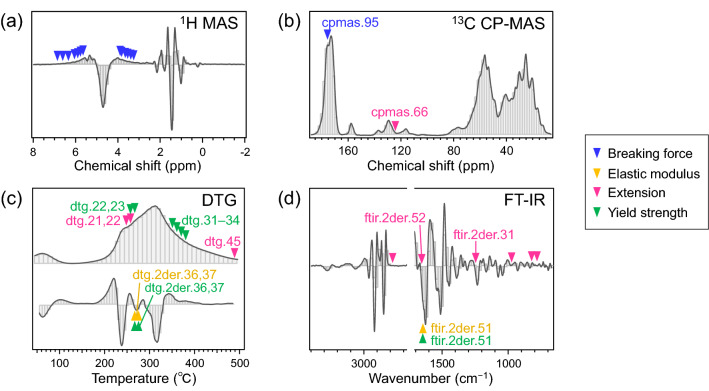
Assignment of measurement descriptors to measured data. The descriptors commonly selected for both RF and PLSR models are shown with arrow heads for breaking force (blue), elastic modulus (orange), extension (pink), and yield strength (green) on (**a**) the second-derivative ^1^H MAS spectrum, (**b**) nonderivative ^13^C CP-MAS NMR spectra, (**c**) nonderivative and second-derivative DTG curves, and (**d**) second-derivative FT-IR spectrum. Symbols of some distinctive descriptors are also indicated with the corresponding colors of the physical properties in each figure.

Based on the descriptors commonly selected by RF and PLSR algorithms up to the 20-th best (Fig. 5), the integrative interpretation of the relationship between the respective physical properties are described. Breaking force selected several descriptors of the ^1^H MAS NMR spectra, around 3.1–3.9 ppm and 5.6–6.8 ppm, which indicate both sides of the peak involving amino acid Hα (blue arrows in Fig. 6a). These signals could be attributed to proteins with strong anisotropic dipolar coupling and, thus, slow mobility. Additionally, the descriptor selected on the ^13^C CP-MAS spectra (“cpmas.95”) corresponds to carbonyl carbons in α-helix form around 176 ppm (Fig. 6b)^9,28,46,47^. The α-helix and coiled-coil structures of crystalline fibrous keratins are distinctive of the cortex component. Therefore, the fraction of rigid α-keratin bundles in the cortex was linked to tensile resistance of hair, as well as the cross-section area. This result also demonstrated that the measurement descriptors successfully represented the secondary structure and the mobility of keratins. Meanwhile, the descriptors of the ^1^H wide-line NMR spectra and TD-NMR were also expected to exhibit molecular mobility; however, they were rarely selected. This result indicated that the descriptors of the ^1^H MAS and ^13^C CP-MAS NMR spectra were substantially efficient because they were well resolved into the spectra and then associated with respective molecular compositions.

The distinctive descriptors selected for elastic modulus were “dtg.2der.36” and “dtg.2der.37” of the DTG curves and “ftir.2der.51” of the FT-IR spectra. “dtg.2der.36” and “dtg.2der.37” correspond to the 265–276 °C range of the second-derivative DTG curves (orange arrows in Fig. 6c). This temperature region could be associated with decomposition of the cuticle, specifically micro-tubes after the cortex has vanished^11,31^. The cow hairs with high elastic modulus showed high, or positive, values for these descriptors, which indicated the relatively slow rate of mass loss in this temperature region. Moreover, “ftir.2der.51” indicates Amide I absorption at 1631–1649 cm^−1^, which is assignable to random coil structure (Fig. 6d)^50–52,59–61^. The FT-IR ATR technique measures only the sample surface, with a depth of several micrometers. Thus, “ftir.2der.51” supposedly corresponds to amorphous keratins in cuticles. Meanwhile, fibrous crystal keratins in cortex remain in α-helical forms during elongation from zero to several percent for evaluating elastic modulus based on Hooke’s law^14,62–64^. Therefore, we assumed that elastic modulus is dependent on the amount of disulfide links or the entanglement of amorphous keratin in the cuticle, rather than the cortex.

Extension was associated with some descriptors of DTG curves (“dtg.21,” “dtg.22,” and “dtg.45”) (Fig. 6c), FT-IR spectra (Fig. 6d), and ^13^C CP-MAS NMR spectra (“cpmas.66”) (Fig. 6b). The referred range (244–263 °C) of the DTG curves in the aforementioned descriptors is possibly related to loss of bound water in the cuticle. “ftir.2der.52” and “ftir.2der.31” represent peaks of Amide I and Amide III at 1651–1669 and 1246–1264 cm^−1^, respectively (pink arrows in Fig. 6d). These regions are assignable to β-turn or random coil structures^50–52,59–61,65^. At the same time, extension of hair reportedly increases with humidity^14,64^. Thus, the selected descriptors potentially demonstrated that the nonorganized amorphous keratins in the cuticle provided accessibility to water and then enhanced the hair extension. The other regions selected on the FT-IR spectra were 2763–2781 (“ftir.2der.60”), 976–993 (“ftir.2der.17”), 822–839 (“ftir.2der.9”), and 783–800 cm^−1^ (“ftir.2der.7”). Although the assignments were difficult, these descriptors possibly represent hydrophilic groups (e.g., C–O and N–H) in proteins that are related to water association. “cpmas.66” is a signal around 124 ppm in the ^13^C CP-MAS NMR spectra, which may result from hydrophilic aromatic amino acids, such as tyrosine. “dtg.45” is also difficult to understand, but could represent carbonization of heat-resistant compositions.

Lastly, yield strength was considerably dependent on the descriptors of the DTG curves (Fig. 6c) and the FT-IR spectra (Fig. 6d). Some of the selected descriptors (i.e., “dtg.2der.36,” “dtg.2der.37,” and “ftir.2der.51”) were common with elastic modulus, which was consistent with the CCorA results (Fig. 3 and Fig. S8). “dtg.22” and “dtg.23” represent the 254–273 °C range on the DTG curve and almost cover the regions of “dtg.2der.36” and “dtg.2der.37.” “dtg.31”–“dtg.34” for the 344–383 °C range were distinctive for yield strength (green arrows in Fig. 6c), which was higher for cat hair and lower for cow and pig hairs. These descriptors presumably indicate highly heat-resistant components in cuticle layers, which induce brittleness in hair.

The prediction accuracies for elastic modulus, extension, and yield strength were not high compared with those for the breaking force (Table 1). This result indicates that elastic modulus, extension, and yield strength need additional information to be sufficiently described. At the same time, errors of evaluated physical property values, which were not considered in model building, possibly hindered to achieve higher prediction accuracies. Nevertheless, the measurement descriptors and the selection strategy demonstrated in the present study successfully provided perspective on relationships with the respective physical properties. In addition, other selected descriptors, which were not discussed above, may support the interpretation of the physical properties. The improvement of model performance can be expected by enriching the variety of hair donors and increasing the repetition number of physical property testing and measurements by respective analytical techniques. Furthermore, importance evaluation and selection of descriptors can be performed using other modeling algorithms^66^. These are worth further detailed investigation in the future.

As for data processing, differentiation was effective to enhance the features of overlapped or broad signals, particularly in the ^1^H MAS NMR spectra and the DTG curves. Moreover, the measurement descriptors selected above were mostly generated by binning rather than dimension reduction and curve deconvolution. This is because binning enables the compression of the measured information more specifically for certain molecular structures, dynamics, and experimental events. At the same time, there have been alternative methods of dimension reduction and deconvolution such as independent component analysis^67^, nonnegative matrix factorization^68^, and t-distributed stochastic neighbor embedding^69^, which may be useful for more efficient extraction of measured information than PCA. Further investigation of data-processing techniques would contribute to the development of descriptors with more efficient and potential compositional information.

## Conclusions

The associations of multiple measured data of hair with its physical properties was investigated by developing a variety of measurement descriptors with different compositional information and by building prediction models based on machine-learning approaches. Descriptor selection based on the “importance” evaluation discovered the most contributive ones for physical property prediction. This then allowed an integrative interpretation of the corresponding relationship based on the manifold measured information: the α-helix and coiled-coil keratins in cortex for breaking force, amorphous keratins or heat-resistant components in cuticle for elastic modulus and yield strength, and water bound to amorphous keratins in cuticle for extension. The results demonstrated the promise of the analytical strategy used in the current study: to associate the various measured information selectively, even if they contribute only partially and even in the presence of substantial errors in objective variables (i.e., physical properties). This analytical strategy will also be applicable to explore compositional information related to other properties of hair for cosmetic purposes; and physicochemical properties of industrial materials composed of cellulose and synthesized polymers with additives, and so on. Further investigation for integrating various measurement data will provide novel and detailed perspectives to comprehensively understand the nature of sophisticated subjects, such as listed above.

## Supplementary Information

Supplementary Information.

## Data Availability

The datasets generated during and/or analyzed during the current study are available from the corresponding author on reasonable request.
