# Supplementary material for: Integrative measurement analysis via machine learning descriptor selection for investigating physical properties of biopolymers in hairs
Source: Sci Rep. 2021 Dec 21;11:24359. doi: 10.1038/s41598-021-03793-9 (PMC8692616; doi:10.1038/s41598-021-03793-9)
Supplement: Supplementary file 1 — Supplementary Information. [file 41598_2021_3793_MOESM1_ESM.pdf]

Supplementary Information

Integrative measurement analysis via machine learning descriptor selection for investigating physical properties of biopolymers in hairs

Ayari Takamura[1], Kaede Tsukamoto[2], Kenji Sakata[1], Jun Kikuchi[1,2,3*]

[1] RIKEN Center for Sustainable Resource Science
1-7-22 Suehiro-cho, Tsurumi-ku, Yokohama, Kanagawa 230-0045, Japan
[2] Graduate School of Medical Life Science, Yokohama City University
1-7-29 Suehiro-cho, Tsurumi-ku, Yokohama, Kanagawa 230-0045, Japan
[3] Graduate School of Bioagricultural Sciences, Nagoya University
1 Furo-cho, Chikusa-ku, Nagoya, Aichi 464-0810, Japan

Correspondence should be addressed to:
Jun Kikuchi (*)
E-mail: jun.kikuchi@riken.jp
Tel.: +81-045-508-7549, Fax: +81-045-503-9489

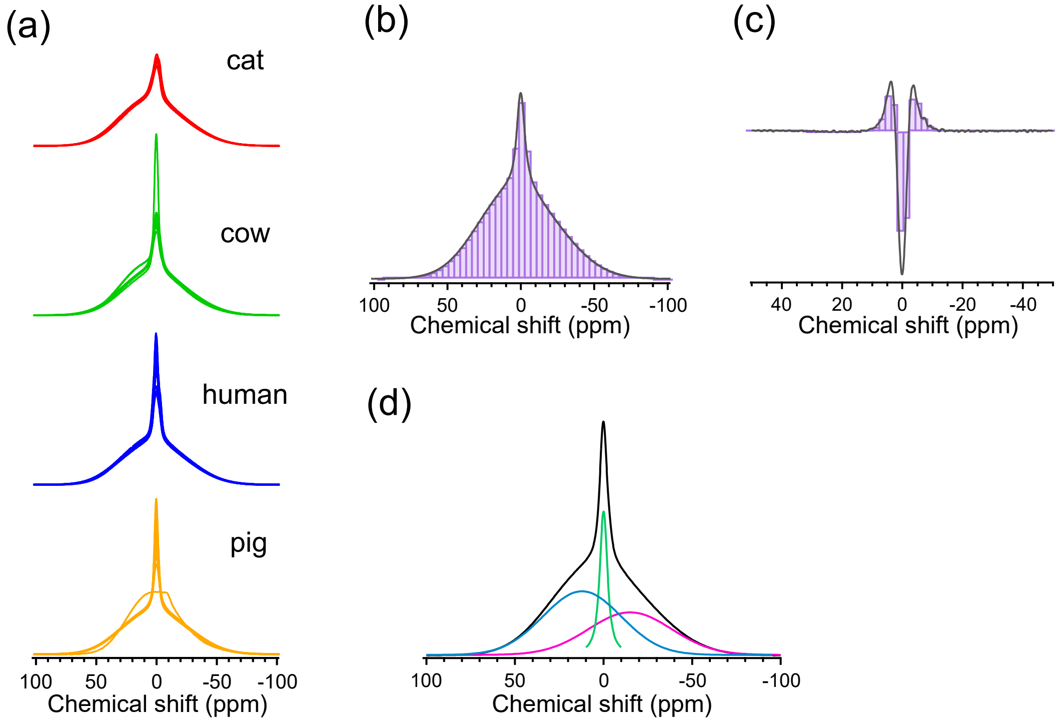

Figure S1. ^{1}H wide-line NMR spectra for generation of measurement descriptors. (**a**) Overlaid ^{1}H wide-line NMR spectra of cat (red), cow (green), human (blue), and pig (orange) hairs. Each spectrum was normalized by the total area. Bins for the (**b**) nonderivative and (**c**) second-order derivative ^{1}H wide-line NMR spectra. The original spectra are drawn with gray lines, and the generated bins are indicated by purple bars. (**d**) Curve deconvolution for ^{1}H wide-line NMR spectra. The original spectrum (gray line) was decomposed into three peaks (magenta, cyan, and green lines) using Voigt functions.

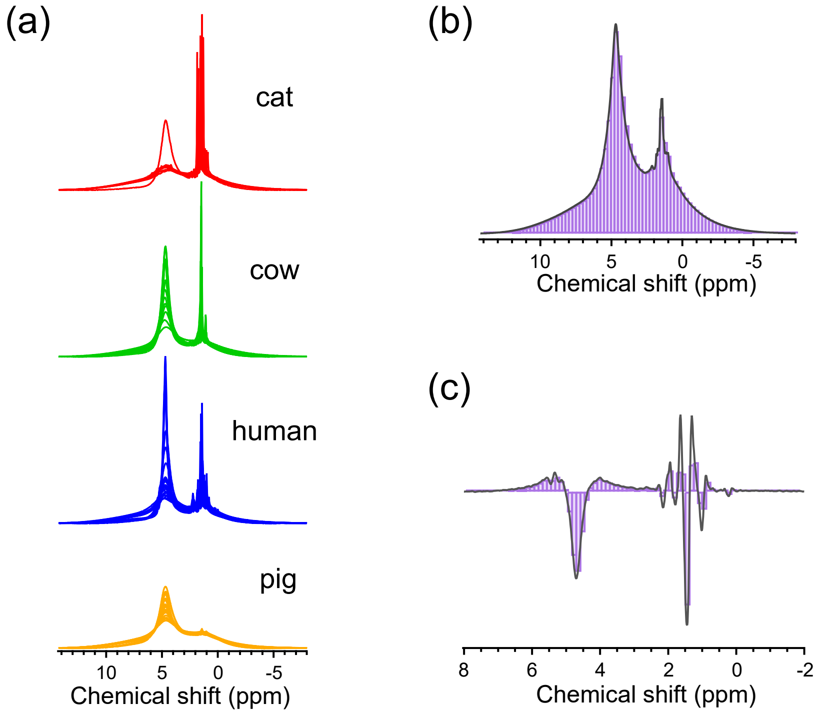

Figure S2. ^{1}H MAS NMR spectra for generation of measurement descriptors. (**a**) Overlaid ^{1}H MAS NMR spectra of cat (red), cow (green), human (blue), and pig (orange) hairs. Each spectrum was normalized by the total area. Bins for the (**b**) nonderivative and (**c**) second-order derivative ^{1}H MAS NMR spectra. The original spectra were drawn with gray lines, and the generated bins are indicated by purple bars.

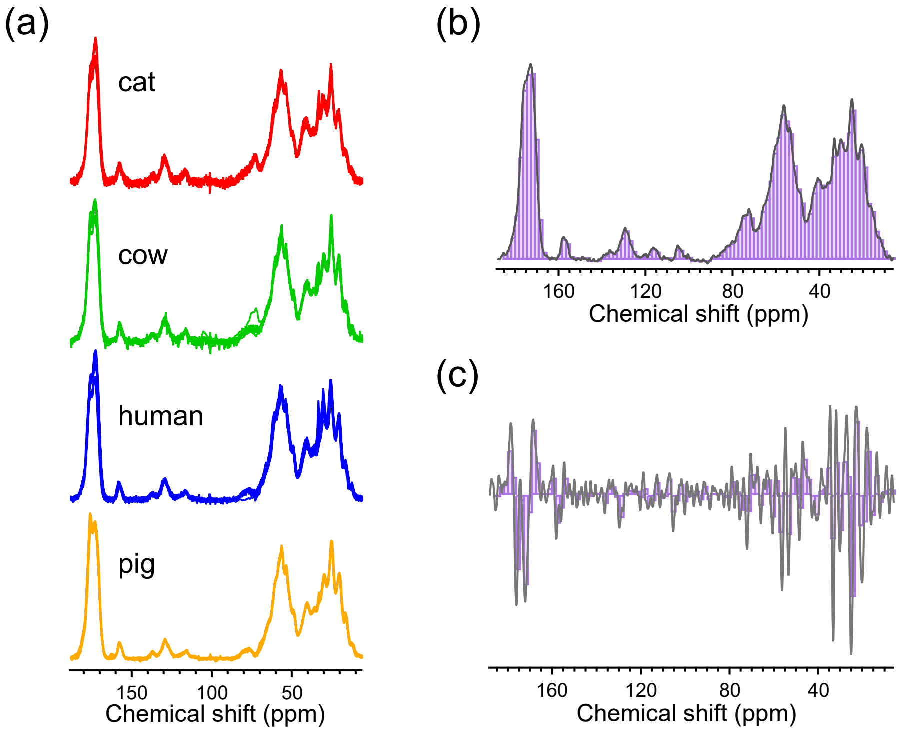

Figure S3. ^{13}C CP-MAS NMR spectra for generation of measurement descriptors. (**a**) Overlaid ^{13}C CP-MAS NMR spectra of cat (red), cow (green), human (blue), and pig (orange) hairs. Each spectrum was normalized by the total area. Bins for the (**b**) nonderivative and (**c**) second-order derivative ^{13}C CP-MAS NMR spectra. The original spectra were drawn with gray lines, and the generated bins are indicated by purple bars.

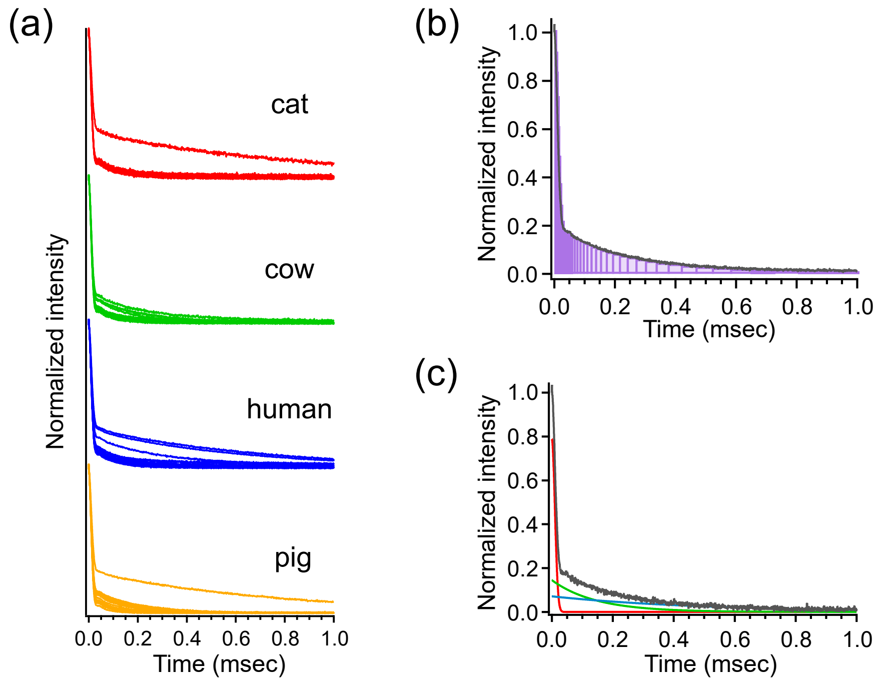

Figure S4. TD-NMR decay curves for generation of measurement descriptors. (**a**) Overlaid TD-NMR decay curves of cat (red), cow (green), human (blue), and pig (orange) hairs. Each spectrum was normalized with an intensity of one at time equal to zero. (**b**) Bins for decay curve of TD-NMR. The original curve was drawn with a gray line, and the generated bins are indicated by purple bars. (**c**) Curve deconvolution for TD-NMR decay curves. The original curve (gray line) was fitted with three components (red, blue, and green lines) using Abragamian functions.

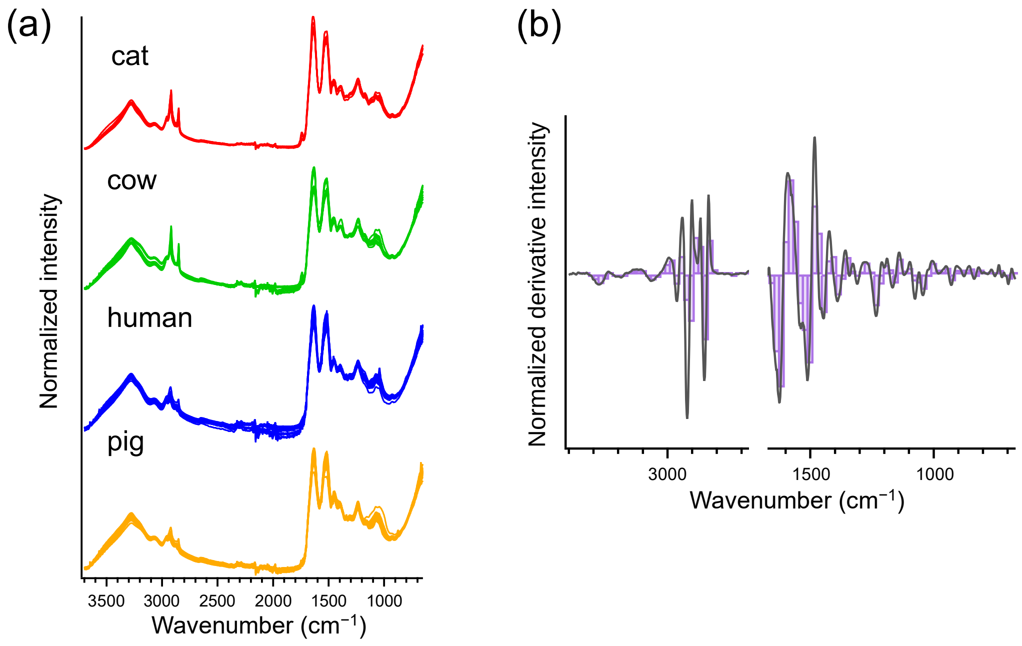

Figure S5. FT-IR spectra for generation of measurement descriptors. (**a**) Overlaid FT-IR spectra of cat (red), cow (green), human (blue), and pig (orange) hairs. Each spectrum was normalized by the total area. (**b**) Bins for the second-order derivative ^{13}C CP-MAS NMR spectra. The original spectra were drawn with gray lines, and the generated bins are indicated by purple bars.

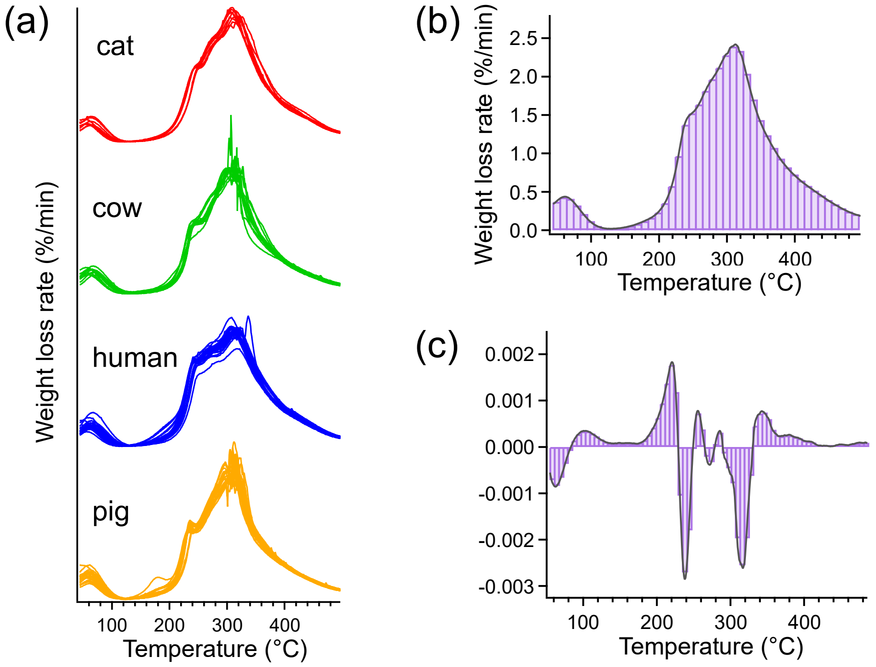

Figure S6. DTG curves for generation of measurement descriptors. (**a**) Overlaid DTG curves of cat (red), cow (green), human (blue), and pig (orange) hairs. Each curve was normalized by the sample weight. Bins for the (**b**) nonderivative and (**c**) second-order derivative DTG curves. The original curves were drawn with gray lines, and the generated bins are indicated by purple bars.

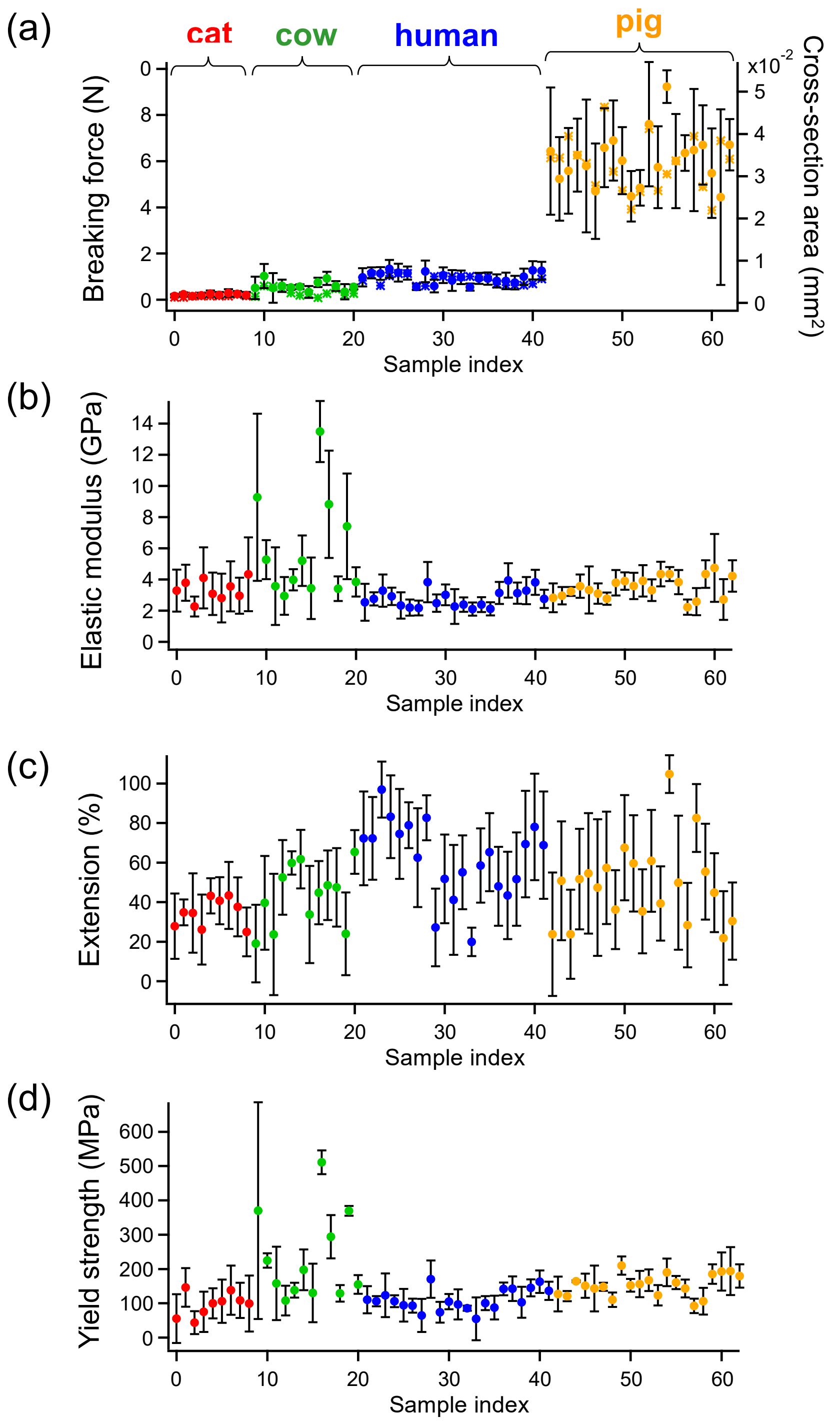

Figure S7. Physical properties of hairs. Hair samples were subjected to a tensile test, and mechanical properties of (**a**) breaking force, (**b**) elastic modulus, (**c**) extension, and (**d**) yield strength were evaluated. Values averaged via testing ten hair fibers are plotted with dots for each donor: cat (red), cow (green), human (blue), and pig (orange). Error bars indicate the standard deviations. The areas of hair cross-sections (stars) are also plotted in (**a**) to show the correlations with breaking force.

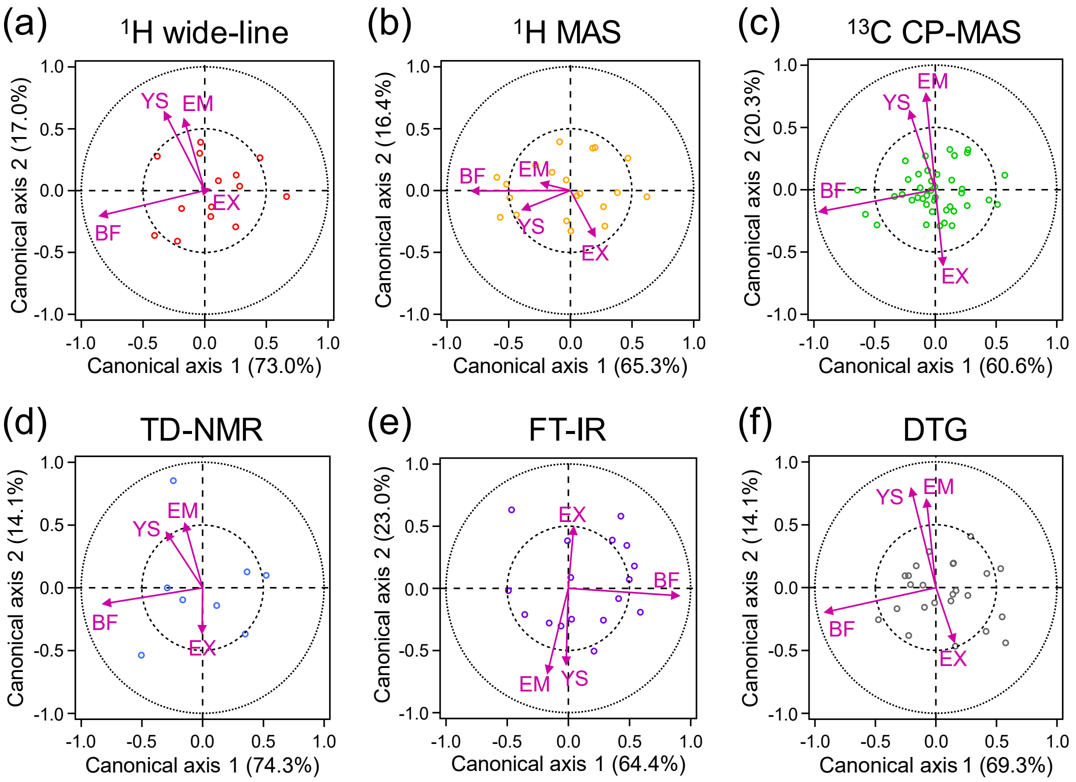

Figure S8. Canonical correlation analysis between physical properties and descriptors from respective measurements of hairs. Datasets of measurement descriptors were prepared from (**a**) ^{1}H wide-line, (**b**) ^{1}H MAS, and (**c**) ^{13}C CP-MAS NMR spectra; (**d**) TD-NMR and (**e**) FT-IR spectra; and (**f**) DTG curves with correlation less than 0.4. Datasets of the physical properties and measurement descriptors were standardized in advance. Scores for the physical properties of breaking force (BF), elastic modulus (EM), extension (EX), and yield strength (YS) are represented with solid arrows.

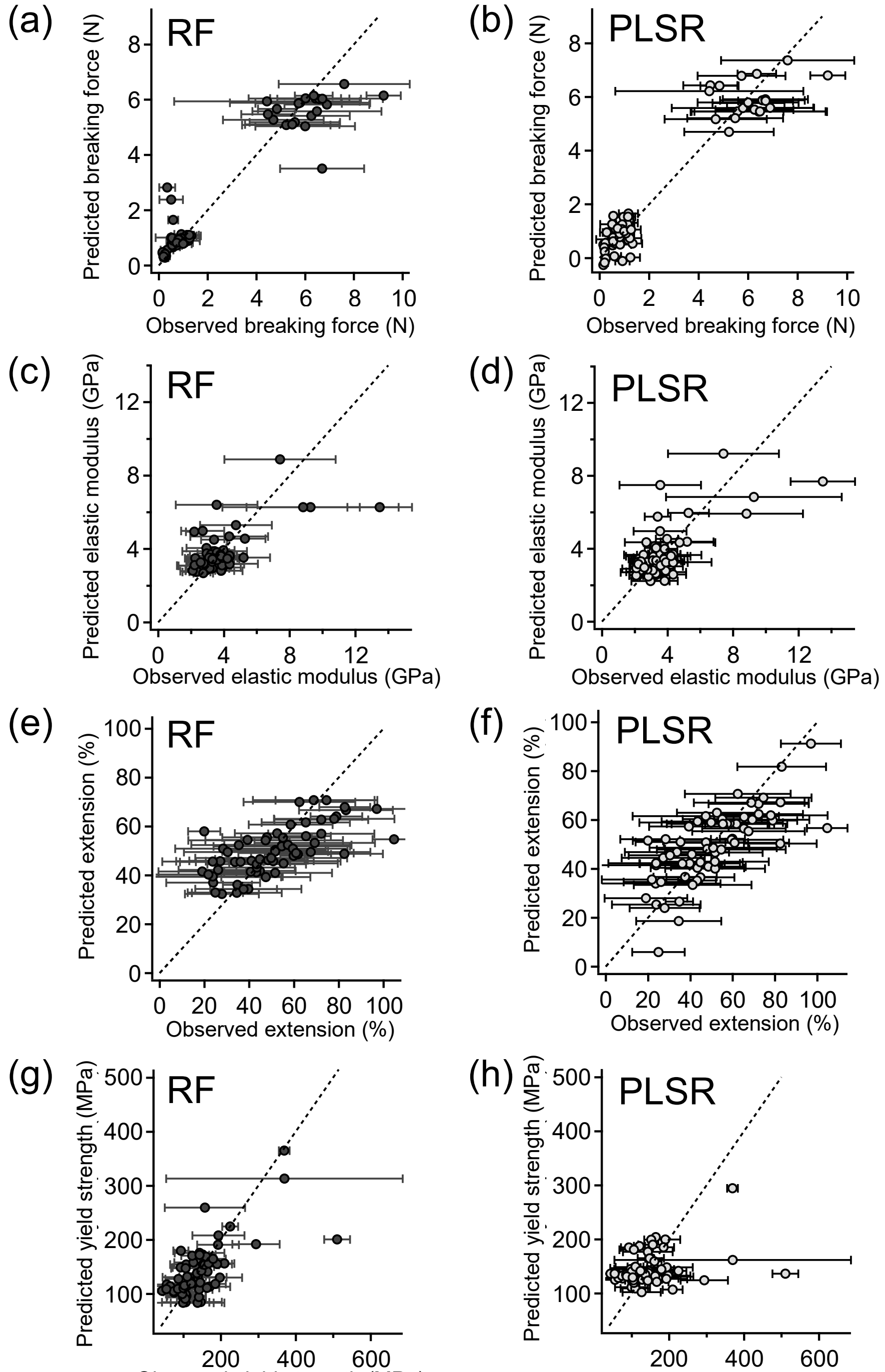

Figure S9. Correlations between observed and predicted physical properties by selected contributive descriptors. The predicted values by one repeat of ten-fold CV with the best descriptor sets were plotted against observed values for (**a,b**) breaking force, (**c,d**) elastic modulus, (**e,f**) extension, and (**g,h**) yield strength in use of (**a,c,e,g**) random forest (RF) and (**b,d,f,h**) partial least squares regression (PLSR) algorithms. Error bars represent standard deviations of ten times of the physical property testing. Broken lines indicate ideal predictions with residual = 0.

Table S1. List of generated measurement descriptors.

Symbols of measurement descriptors	Data information	Count
Solid state-NMR/^{1}H wide-line (anisotropic) spectra		
H.ani.1, H.ani.2, …, H.ani.51	Bins of nonderivative ^{1}H wide-line spectra at −102–102 ppm	51
H.ani.2der.1, H.ani.2der.2, …, H.ani.2der.51	Bins of second-order derivative ^{1}H wide-line spectra at −51–51 ppm	51
S.ani.1, S.ani.2, …, S.ani.6	Scores of 1st–6th PCs of nonderivative ^{1}H wide-line spectra	6
S.ani.2der.1, S.ani.2der.2, …, S.ani.2der.8	Scores of 1st–8th PCs of second-order derivative ^{1}H wide-line spectra	8
A1.ani, A2.ani, A3.ani	Area proportions of decomposed peaks	3
iA1.ani, iA2.ani, iA3.ani	Inverses of area proportions of decomposed peaks	3
log.A1.ani, log.A2.ani, log.A3.ani	Logarithms of area proportions of decomposed peaks	3
exp.A1.ani, exp.A2.ani, exp.A3.ani	Exponentials of area proportions of decomposed peaks	3
exp.iA1.ani, exp.iA2.ani, exp.iA3.ani	Exponentials of inverses of area proportions of decomposed peaks	3
A1.ani/A2.ani, A1.ani/A3.ani, A2.ani/A1.ani, A2.ani/A3.ani, A3.ani/A1.ani, A3.ani/A2.ani	Mutual ratios of area proportions of decomposed peaks	6
FWHM1.ani, FWHM2.ani, FWHM3.ani	FWHM (ppm) of decomposed peaks	3
iFWHM1.ani, iFWHM2.ani, iFWHM3.ani	Inverses of FWHM of decomposed peaks	3
log.FWHM1.ani, log.FWHM2.ani, log.FWHM3.ani	Logarithms of FWHM of decomposed peaks	3
exp.FWHM1.ani, exp.FWHM2.ani, exp.FWHM3.ani	Exponentials of FWHM of decomposed peaks	3
exp.iFWHM1.ani, exp.iFWHM2.ani, exp.iFWHM3.ani	Exponentials of inverses of FWHM of decomposed peaks	3
FWHM1.ani/FWHM2.ani, FWHM1.ani/FWHM3.ani, FWHM2.ani/FWHM1.ani, FWHM2.ani/FWHM3.ani, FWHM3.ani/FWHM1.ani, FWHM3.ani/FWHM2.ani	Mutual ratios of FWHM of decomposed peaks	6
Solid state-NMR/^{1}H MAS (isotropic) spectra		
H.iso.1, H.iso.2, …, H.iso.90	Bins of nonderivative ^{1}H MAS spectra at −8.0–14.2 ppm	90
H.iso.2der.1, H.iso.2der.2, …, H.iso.2der.80	Bins of second-order derivative ^{1}H MAS spectra at −1.8–8.0 ppm	80
S.iso.1, S.iso.2, …, S.iso.9	Scores of 1st–9th PCs of nonderivative ^{1}H MAS spectra	9
S.iso.2der.1, S.iso.2der.2, …, S.iso.2der.9	Scores of 1st–9th PCs of second-order derivative ^{1}H MAS spectra	9
Solid state-NMR/CP-MAS spectra		
cpmas.1, cpmas.2, …, cpmas.101	Bins of nonderivative CP-MAS spectra at 2.8–185.2 ppm	101
cpmas.2der.1, cpmas.2der.2, …, cpmas.2der.101	Bins of second-order derivative CP-MAS spectra at 2.8–185.2 ppm	101
S.cpmas.1, S.cpmas.2, …, S.cpmas.10	Scores of 1st–10th PCs of nonderivative CP-MAS spectra	10
S.cpmas.2der.1, S.cpmas.2der.2, …, S.cpmas.2der.16	Scores of 1st–16th PCs of second-order derivative CP-MAS spectra	16

Table S1. (continued)

Time-domain NMR		
td.1, td.2, …, td.40	Bins of TD-NMR decay curves with logarithmic steps	40
S.td.1, S.td.2	Scores of 1st and 2nd PCs of decay curves	2
S.td.comp1.1, S.td.comp1.2, S.td.comp1.3	Scores of 1st–3rd PCs of decay curves fitted for the first component	3
S.td.comp2.1, S.td.comp2.2, S.td.comp2.3	Scores of 1st–3rd PCs of decay curves fitted for the second component	3
S.td.comp3.1, S.td.comp3.2, S.td.comp3.3	Scores of 1st–3rd PCs of decay curves fitted for the third component	3
S.td.comp123.1, S.td.comp123.2, …, S.td.comp123.4	Scores of 1st–4th PCs of the three fitted decay curves, concatenated in tandem	4
A1.td, A2.td, A3.td	Intensity proportions of the three fitted components	3
iA1.td, iA2.td, iA3.td	Inverses of intensity proportions of the three fitted components	3
log.A1.td, log.A2.td, log.A3.td	Logarithms of intensity proportions of the three fitted components	3
exp.A1.td, exp.A2.td, exp.A3.td	Exponentials of intensity proportions of the three fitted components	3
A1.td/A2.td, A1.td/A3.td, A2.td/A1.td, A2.td/A3.td, A3.td/A1.td, A3.td/A2.td	Mutual ratios of intensity proportions of the three fitted components	6
T1.td, T2.td, T3.td	Relaxation times (ms) of the three fitted components	3
iT1.td, iT2.td, iT3.td	Inverses of relaxation times (ms) of the three fitted components	3
log.T1.td, log.T2.td, log.T3.td	Logarithms of relaxation times (ms) of the three fitted components	3
exp.T1.td, exp.T2.td, exp.T3.td	Exponentials of relaxation times (ms) of the three fitted components	3
T1.td/T2.td, T1.td/T3.td, T2.td/T1.td, T2.td/T3.td, T3.td/T1.td, T3.td/T2.td	Mutual ratios of relaxation times of the three fitted components	6
FT-IR		
ftir.2der.1, ftirr.2der.2, …, ftirr.2der.91	Bins of second-order derivative FT-IR spectra at 667–3400 cm^{-1} except 1711–2669 cm^{-1}	91
S.ftir.2der.1, S.ftir.2der.2, …, S.ftir.2der.9	Scores of 1st–9th PCs of second-order derivative FT-IR spectra	9
TG-DTA		
dtg.1, dtg.2, …, dtg.45	Bins of DTG curves at 44°C–497°C	45
dtg.2der.1, dtg.2der.2, …, dtg.2der.72	Bins of second-order derivative DTG curves at 57°C–486°C	72
S.dtg.1, S.dtg.2, …, S.dtg.11	Scores of 1st–11th PCs of DTG curves	11
S.dtg.2der.1, S.dtg.2der.2, …, S.dtg.2der.9	Scores of 1st–9th PCs of second-order derivative DTG curves	9
	Total	902

Table S2. Prediction of physical properties by all descriptors generated from respective measurements via RF or PLSR algorithms.[a]

Modeling algorithm	Measurement (input)	Physical property (output)							
		Breaking force (N)		Elastic modulus (GPa)		Extension (%)		Yield strength (MPa)	
		RMSE	R^2	RMSE	R^2	RMSE	R^2	RMSE	R^2
RF	^1H wide-line	1.37 ± 0.04	0.761 ± 0.018	1.80 ± 0.06	0.107 ± 0.031	19.7 ± 0.4	0.032 ± 0.018	70.1 ± 3.8	0.173 ± 0.060
	^1H MAS	0.91 ± 0.02	0.882 ± 0.007	1.88 ± 0.04	0.030 ± 0.011	19.8 ± 0.4	0.017 ± 0.015	75.3 ± 1.5	0.065 ± 0.019
	^{13}C CP-MAS	1.01 ± 0.02	0.885 ± 0.007	1.79 ± 0.03	0.052 ± 0.019	20.0 ± 0.3	0.009 ± 0.011	75.7 ± 1.2	0.030 ± 0.019
	TD-NMR	1.27 ± 0.04	0.765 ± 0.014	1.91 ± 0.06	0.031 ± 0.017	21.6 ± 0.4	0.010 ± 0.009	76.0 ± 2.0	0.072 ± 0.022
	FT-IR	1.08 ± 0.02	0.864 ± 0.008	1.59 ± 0.04	0.251 ± 0.039	18.0 ± 0.4	0.170 ± 0.028	64.0 ± 1.9	0.296 ± 0.042
	DTG	1.28 ± 0.03	0.805 ± 0.013	1.60 ± 0.06	0.240 ± 0.058	18.8 ± 0.3	0.098 ± 0.019	59.5 ± 2.2	0.402 ± 0.048
	All	0.85 ± 0.02	0.913 ± 0.005	1.65 ± 0.03	0.204 ± 0.039	18.1 ± 0.3	0.162 ± 0.038	65.6 ± 1.5	0.309 ± 0.048
PLSR	^1H wide-line	1.58 ± 0.05	0.647 ± 0.019	1.87 ± 0.08	0.141 ± 0.040	19.7 ± 0.2	0.026 ± 0.008	75.7 ± 5.1	0.141 ± 0.052
	^1H MAS	1.06 ± 0.04	0.838 ± 0.012	1.85 ± 0.03	0.023 ± 0.010	20.4 ± 0.4	0.029 ± 0.016	73.4 ± 2.8	0.162 ± 0.039
	^{13}C CP-MAS	0.96 ± 0.03	0.866 ± 0.009	1.92 ± 0.05	0.028 ± 0.015	18.6 ± 0.3	0.127 ± 0.021	80.1 ± 2.9	0.043 ± 0.023
	TD-NMR	1.68 ± 0.10	0.615 ± 0.032	1.87 ± 0.05	0.033 ± 0.016	20.7 ± 0.4	0.011 ± 0.018	78.4 ± 3.5	0.154 ± 0.035
	FT-IR	1.26 ± 0.03	0.773 ± 0.011	1.68 ± 0.04	0.202 ± 0.028	18.1 ± 0.2	0.164 ± 0.016	66.3 ± 1.7	0.274 ± 0.028
	DTG	1.30 ± 0.04	0.756 ± 0.016	1.56 ± 0.06	0.292 ± 0.049	19.7 ± 0.4	0.088 ± 0.021	58.6 ± 1.9	0.409 ± 0.037
	All	0.81 ± 0.03	0.906 ± 0.007	1.61 ± 0.04	0.254 ± 0.033	17.5 ± 0.4	0.233 ± 0.028	62.2 ± 2.2	0.336 ± 0.046

[a] Each value was evaluated by 100 repeats of ten-fold CV (average ± standard deviation).
